# Improved corrosion resistance of permanganate-phosphate conversion coat on steel surface by surfactants

**DOI:** 10.1038/s41598-023-41394-w

**Published:** 2023-09-22

**Authors:** B. A. Abd-El-Nabey, S. El-Housseiny, M. A. Abd-El-Fatah

**Affiliations:** 1 Chemistry Department, Faculty of Science, Alexandria University, Alexandria, Egypt; 2 Chemistry Department, Faculty of Sciences, Jouf University, Sakaka, Saudi Arabia; 3 Chemistry Department, Faculty of Science, Kuwait University, Kuwait City, Kuwait; 4 Faculty of Education, Alexandria University, Alexandria, Egypt

**Keywords:** Chemistry, Materials science

## Abstract

In the present work, we studied the effect of the presence of different concentrations of each of Triton-X-100 and Tween-80 surfactants in the bath of permanganate-phosphate conversion coating (PPC) on the corrosion resistance and the microstructure of the prepared coats. The coats were investigated using a scanning electron microscope (SEM), an energy dispersive X-ray spectrometer (EDX), X-ray photoelectron spectroscopy (XPS), electrochemical impedance spectroscopy (EIS), and potentiodynamic polarization techniques. The SEM results show that, on addition of the surfactants to the PPC bath, the porosity of the coat decreases and the coating layer becomes more compact. EIS results indicated that the presence of 0.01 M Triton-X-100 or 0.01 M Tween-80 in the coating solution caused an increase in the protection efficiency of the coat up to 93.7% and 84.1%, respectively. The potentiodynamic polarization results indicated that the two surfactants mainly act as anodic inhibitors due to the adsorption of their molecules at the anodic sites of the surface of steel and retard its oxidation reaction. The EDX and XPS results confirmed the results of the other techniques. A mechanism for the role of the surfactants in the coating process was proposed using the results of XPS and the other techniques.

## Introduction

Mild steel is used in many industries, such as metallurgy, automobiles, airspace, shipbuilding sectors, and the chemical industry^1–3^. However, its susceptibility to corrosion in several environments limits its applications. Ammonium nitrate is used as agricultural fertiliser. During its manufacture, the used carbon steel reactor suffered severe corrosion^4^. Conversion coatings are one of the most successful strategies for preventing steel from corroding, among other methods^5–8^.

Chromate conversion coating is extensively used to prevent corrosion in metals. Because Cr (VI) is a carcinogen, chromate conversion coating methods have been alleviated by several international directives^9^. The development of environmentally friendly techniques for free -chromate conversion coats is now all the more important, such as one based on phosphate^10–13^, molybdates^14–18^, and rare-earth (cerium) metal salts^19–23^. Due to the chemical bonding between the matrix metal and coating, the phosphate chemical conversion coating has excellent binding performance^24,25^. The role of nano ZnO particles in the electrodeposition and growth mechanism of phosphate coating for enhancing the anti- corrosive performance of low carbon steel in a 3.5% NaCl solution was studied by Kathavate et al.^26^. They found that nano ZnO powder promotes the formation of hopeite [Zn_3_ (PO4)_2_–4H_2_O] and phosphophylite [Zn Fe (PO_4_)_2_–4H_2_O] phases which are the main constituents of phosphate_ conversion coatings. Polyethylene glycol was used by Liu et al.^27^ as an additive for zinc phosphating treatment. They found that the growth trend of hopeite increased along (020) and (220) directions, and the crystal growth changed from the axial to the radial directions. When the concentration of polyethylene glycol was 0.8 g/L, the zinc phosphate conversion coating exhibited the most uniform and dense structure and the strongest corrosion resistance. To improve the corrosion protection properties of zinc-rich silicate coatings on steel, zirconium pretreatment loaded with (3- amino propyl) triethoxy silence (APTES) 0.025% (V/V) and the partial replacement of spherical zinc by flake Zn Al alloy were investigated by Hoang et al.^28^. The results showed that flake Zn Al pigment (5 wt%) significantly improved corrosion resistance and prolonged the duration of cathodic protection of zinc- rich silicate coatings. The silane and graphene oxide (GO) nanocomposite was successfully created by Du et al. They then investigated the improved chemical and mechanical performance of the electrodeposited silane/GO nanocomposite on the copper surface. The outcomes showed that after spending 120 h submerged in a 1 mol/L sodium chloride aqueous solution, the protection efficiency was maintained above 99.8%^29^. Chen et al.^30,31^ used ATR-FTIR, Raman, EDS, XPS, and EIS techniques to explore hydrophobic silane/graphene oxide (GO) composite coatings and BTAH inhibitor-silane/GO on copper surface. The findings demonstrated that the coated copper’s corrosion current density decreased by over three orders of magnitude when compared to the copper left naked in a 1 M neutral NaCl solution and remained above 99% even after 120 h of immersion. Additionally, using a simple co-electrodeposition procedure, they were successful in building this nanocomposite on an AA2024-T3 aluminium alloy surface^32^.

Due to their beneficial effects, useful applications, and low cost, surfactants are one of the environmentally friendly corrosion inhibitors that are receiving significant interest and preference^33–35^. It can be used as an effective additive to give the most desirable coatings, because it affects the growth and modification of the conversion coating crystal's microstructure^36,37^. Recently, we studied the effect of each of Tween-80, Cetyl trimethyl ammonium bromide (Cetrimide), and Sodium Lauryl Sulphate (SLS) surfactants on the corrosion resistance, porosity, structure, and composition of the Zn-phosphate coat on steel^38^. Results of potentiodynamic polarization and electrochemical impedance spectroscopy techniques revealed that the addition of 0.01 M surfactants to the coating solution resulted in a reduction in porosity and an elevation in the coat's protection efficiency of roughly 90%. The outcomes demonstrated that the coating process was regulated by the surfactant molecules' adsorption to the steel surface.

The novelty of this work is improving the corrosion resistance of the permanganate–phosphate conversion coats on the surface of steel by two neutral surfactants with different molecular structures, Tween-80 and Triton-X-100. The corrosion resistance of the coats was determined using electrochemical techniques, potentiodynamic polarizations, and electrochemical impedance spectroscopy (EIS). Moreover, the morphology and structure of the coats were investigated using surface techniques, a scanning electron microscope (SEM), an energy dispersive X-ray spectrometer (EDX), and X-ray photoelectron spectroscopy (XPS) techniques. A mechanism of the action of surfactants in the conversion coating process was proposed using the data from the XPS and other techniques.

## Experimental

### Materials and solutions

Mild steel was utilized as a test material. The chemical composition of it was listed in Table 1.

**Table 1:** The Mild steel chemical composition (wt.%).

C	S	Mn	P	Si	Fe
0.21	0.04	2.5	0.04	0.35	Balance

The steel electrode was constructed in a cylindrical shape and covered in epoxy resin such that just one surface with a surface area of 1 cm^2^ was exposed, preventing the crevice effect.

**Figure 1:**
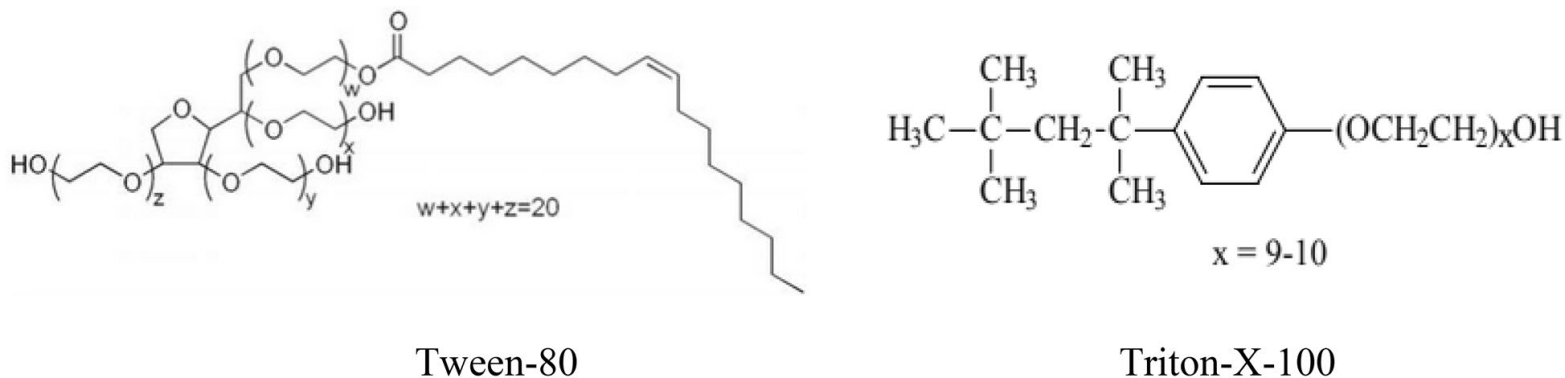
Chemical structure of Tween-80 and Triton-X-100 surfactants.

Distilled water and chemicals of a high caliber were used to create the stock solutions: 85% H_3_PO_4_, KMnO_4_, NaHPO_4_, and NH_4_NO_3_ were acquired from Aldrich chemicals. Tween-80 and Triton-X-100 (Fig. 1) were obtained from Alpha Chemika. Many studies provide valuable information on selection of surfactants in different critical applications. Tween-80 and Triton-X-100 surfactants were found to be safe in low concentrations^39–41^.

### Application of conversion coat

The conversion coating solution was prepared by mixing 0.75 g of NaHPO_4_, 1 g KMnO_4_ and 2 ml H_3_PO_4_ in a 100 ml flask with the addition of a certain volume of the Tween-80 or Triton-X-100 and double distilled water to give the desired surfactant concentration. The pH of the coating solution was maintained at value 3. The steel electrode with area 0.2826 cm^2^ mild steel was used. The steel electrode was pretreated as follows: hand-polishing using emery paper from 400 up to1000 grit, starting with a coarse grit and working up to a fine one till reaching a mirror finish follows by the pickling process by the identical procedure that was reported in the earlier work^38^. For each experiment, A rotator supported the steel electrode (60 rotates per min.) with an immersion time of 30 min. The coat obtained covered all the surface of steel electrode.

**Figure 2:**
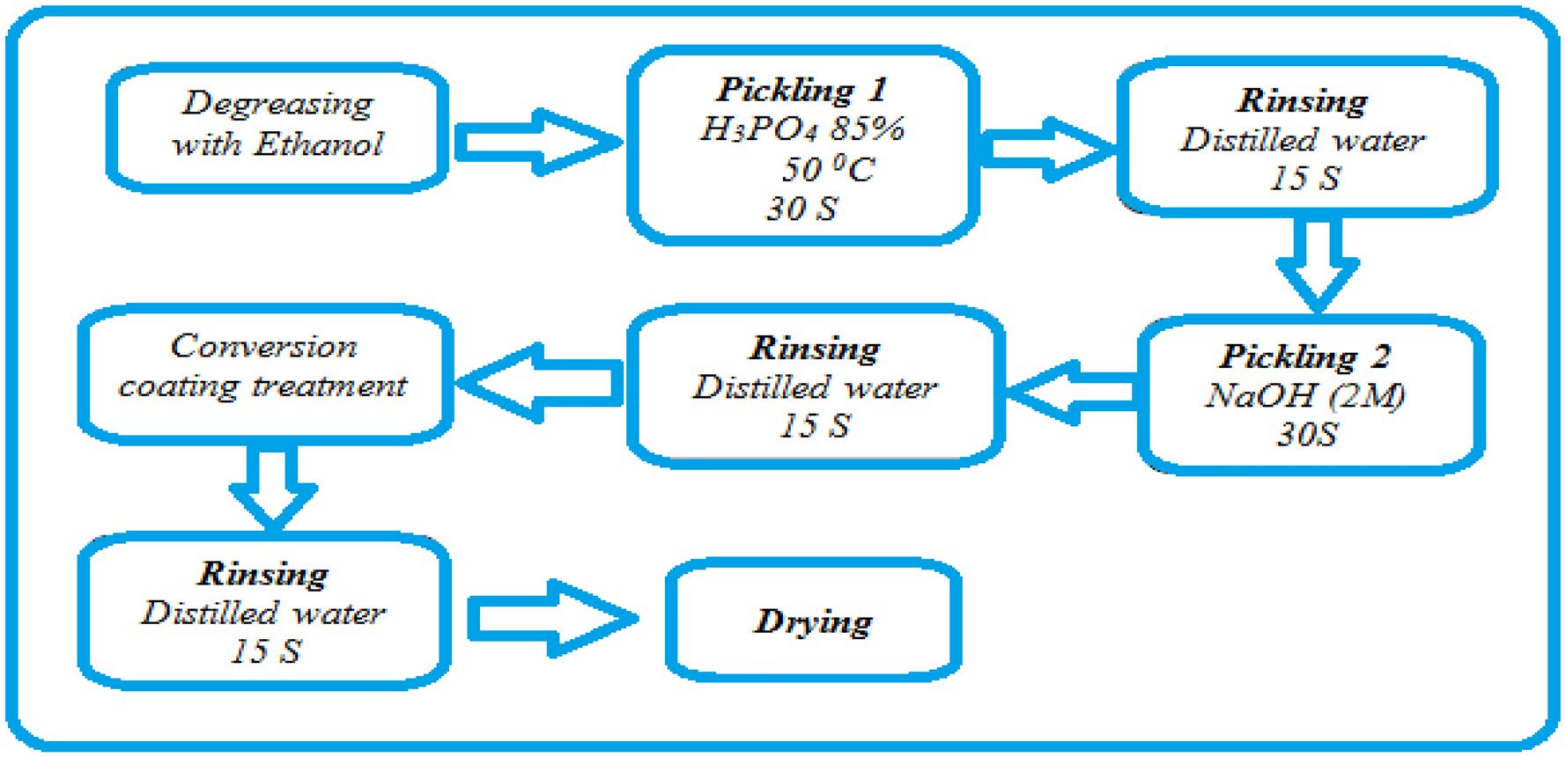
Experimental procedure used for the samples preparation.

Figure 2 provides a detailed description of the treatment procedure including the degreasing process, the phosphoric and soda pickling stage, the conversion treatment, and the final rinse and drying step.

### Microstructural characterization

Scanning electron microscopy (SEM) with the JOEL instrument used to examine the surface morphology of the permanganate-phosphate coating without and with Triton x-100 or Tween-80. Coupons with an area of 1 cm^2^ steel samples were used in the experiments. The elements of the film that were created on the surface were analyzed by an energy-dispersive X-ray spectrometer (EDS, JEM-2100, Japan). Also, Dry film thickness (PosiTest DFT Ferrous) or coating thickness was measured.

### XPS analysis

For X-ray photoelectron spectroscopy (XPS) analysis, 1 cm^2^ metallic specimen, after washing with distilled water (conductivity: 6 × 10^−4 ^Ω^−1^m^−1^) and drying, was placed on a specific sample holder then moved to an ultra-high vacuum chamber at 10^−9 ^torr. The X-ray source (made by VG Microtech, model XR3E2) with Al anode and X-ray energy (1486.6 eV) was used to excite the surface.

### Electrochemical measurements

Without stirring and with air bubbling, measurements of the electrochemical nature were carried in a 100 ml cell using three electrodes, with the working electrode facing the counter electrode. (a graphite rod) and saturated calomel electrodes (SCE) as the reference electrode. In the text, all potentials are provided in respect to this reference electrode. The electrolyte used was 0.6M NH_4_NO_3_. Electrochemical tests were carried out using a frequency response analyzer (FRA)/potentiostat supplied by Parstat Instrument (PARSTAT 2263.02 SN 194). Before performing the electrochemical tests, the working electrode with area 0.2826 cm^2^ was put into a 0.6 M NH_4_NO_3_ solution and left there for 30 min at room temperature to get the rest potential.

Potentiodynamic polarization measurements were operated with a scan rate of 20 mV/min starting from cathodic potential (E_corr_ − 300 mV) going to anodic direction (E_corr_ + 900 mV). And the measurements of the electrochemical impedance spectrum from 3.2 × 10^4^ to 0.1 Hz with 10 mV amplitude around the rest potential. For the consistency of the measurements, each experiment with the same conditions was performed twice and the results had a 2% deviation.

## Results and discussion

### SEM Study and EDX analysis

**Figure 3:**
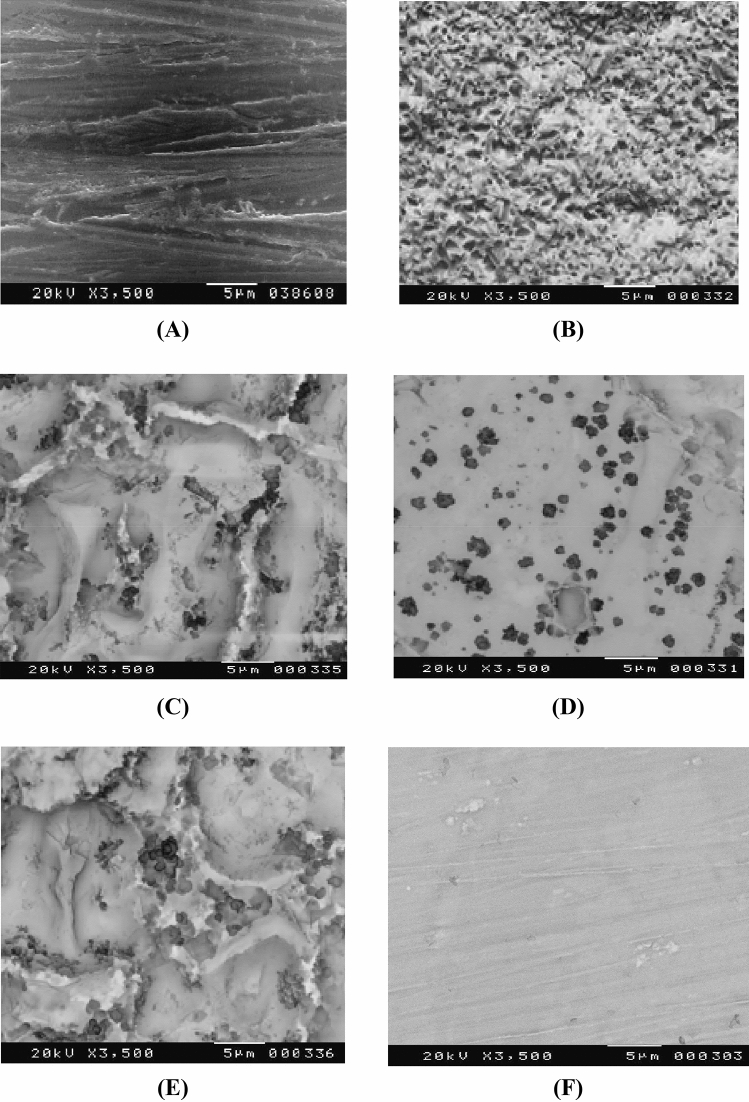
SEM images for mild steel (**A**) Bare steel, (**B**) coated steel, (**C**) coated steel in presence of 5 × 10^−5^M Tween-80, (**D**) coated steel in presence of 5 × 10^−3^M Tween-80, (**E**) coated steel in presence of 5 × 10^−5^M Triton-X-100 and (**F**) coated steel in presence of 5 × 10^−3^M Triton-X-100.

Figure 3 shows the micrographs of SEM graphs of (A): Bare steel, (B): coated steel, (C): coated steel in presence of 5 × 10^−5^M Tween-80, (D): coated steel in presence of 5 × 10^−3^M Tween-80, (E): coated steel in presence of 5 × 10^−5^M Triton-X-100 and (F) coated steel in presence of 5 × 10^−3^M Triton-X-100. It is clear that both the surfaces of the bare steel and the coat B have significant porosity in the absence of surfactants. Upon progressively adding more of the surfactants during the coating process (C, D, E, F), the coating layer becomes more dense and the porosity of the coat diminishes. The SEM images demonstrate that the presence of surfactants reduces the number of surface holes, and that the shape of the surface becomes uniform.

**Figure 4:**
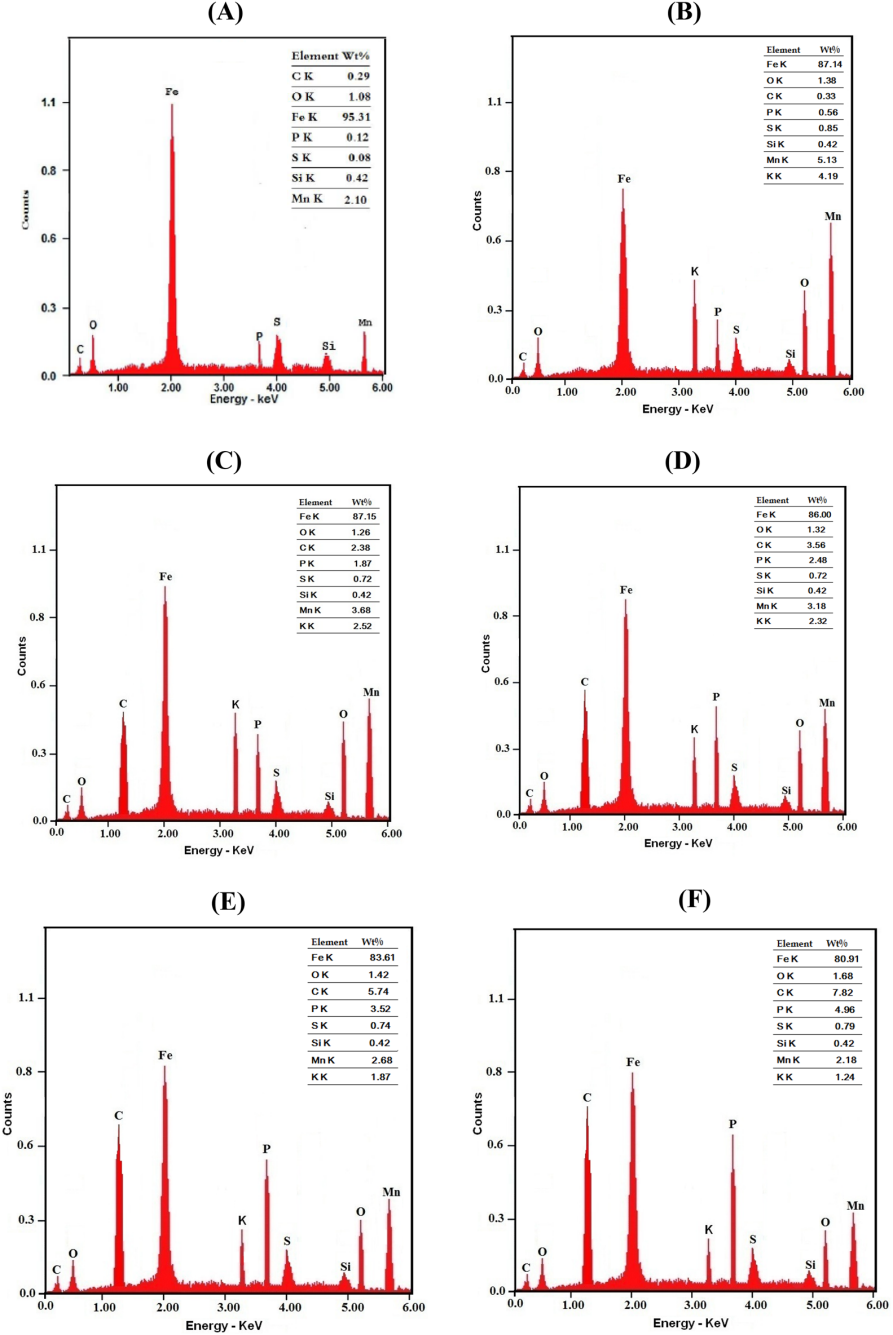

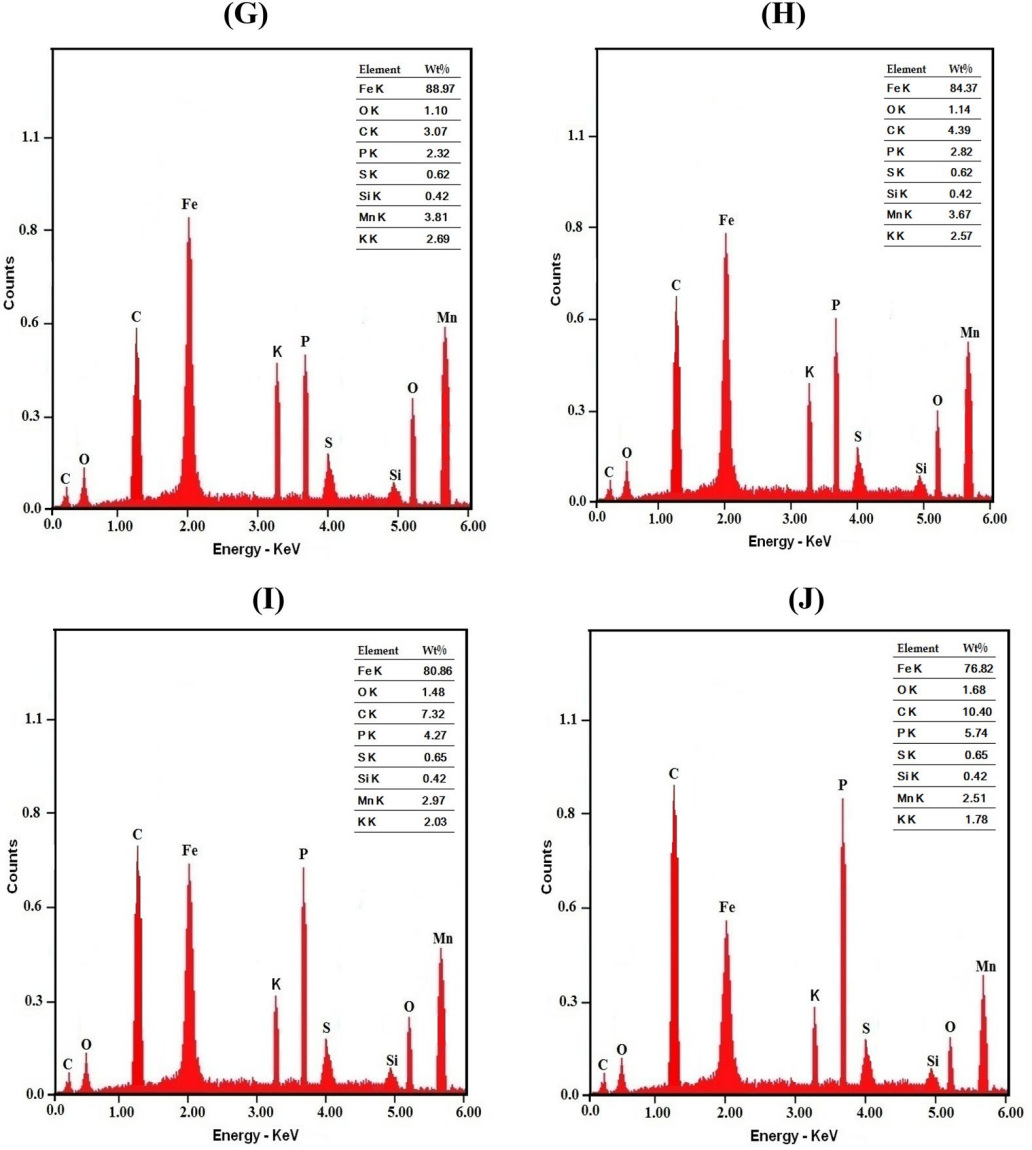
EDX micrographs for mild steel (**A**) Bare steel, (**B**) coated steel, (**C**) coated steel in presence of 1 × 10^−5^M Tween-80, (**D**) coated steel in presence of 5 × 10^−5^M Tween-80, (**E**) coated steel in presence of 5 × 10^−4^M Tween-80, (**F**) coated steel in presence of 5 × 10^−3^ M Tween-80, (**G**) coated steel in presence of 1 × 10^−5^M Triton-X-100, (**H**) coated steel in presence of 5 × 10^−5^M Triton-x-100, (**I**) coated steel in presence of 5 × 10^−4^M Triton-X-100 and (**J**) coated steel in presence of 5 × 10^−3^M Triton-X-100.

**Figure 5:**
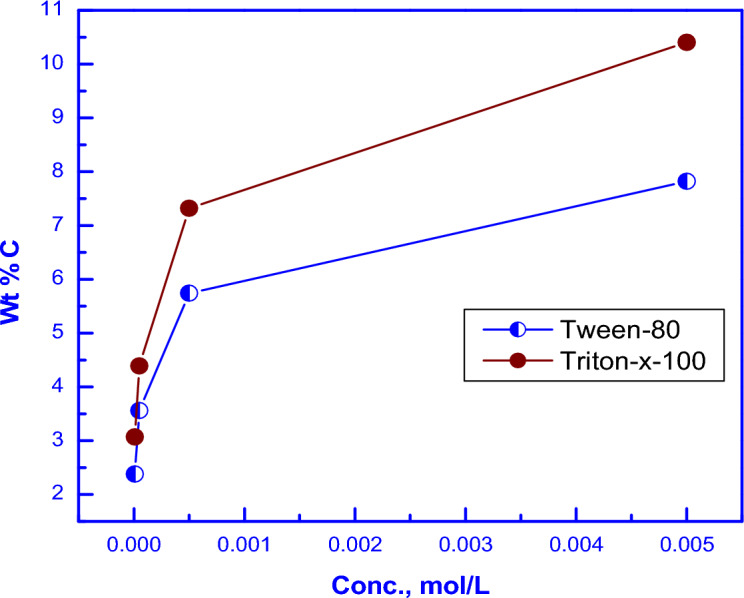
Variations of the weight percent of carbon (Wt % C) on the coated surface with the concentration of each of Triton-X-100 or Tween-80 present in the coating solution.

The EDX results are represented in Fig. 4. Variations of the weight percent of carbon (Wt % C) on the coated surface with the concentration of the surfactants in the coating solution are shown in Fig. 5. It is clear that in presence of low concentrations (≤ 0.0001 M) of the surfactants in the solution, Wt % C on coat surface sharply increases. However, in the presence of higher concentrations in the solution, change of the concentration of the surfactant has a slight effect on its concentration on the surface. This behaviour can be discussed on the basis that in presence of low concentrations of the surfactants in the coating solution, the increase of the amount of the surfactant greatly raises the value of the surface coverage; however, in presence of higher concentrations, the steel surface becomes saturated with adsorbed surfactant molecules. Figure 5 shows also that Wt %C on the coat surface in presence of Triton-X-100 surfactant in the coating bath is higher than that in presence of Tween-80 surfactant. This behaviour can be attributed to the presence of a benzene ring in the molecular structure of the Triton-X-100 molecule which leads to the increase of its π-system and its donation of the electrons to metal surface making it strong absorbable^42,43^.

### Electrochemical impedance spectroscopy (EIS) results

**Figure 6:**
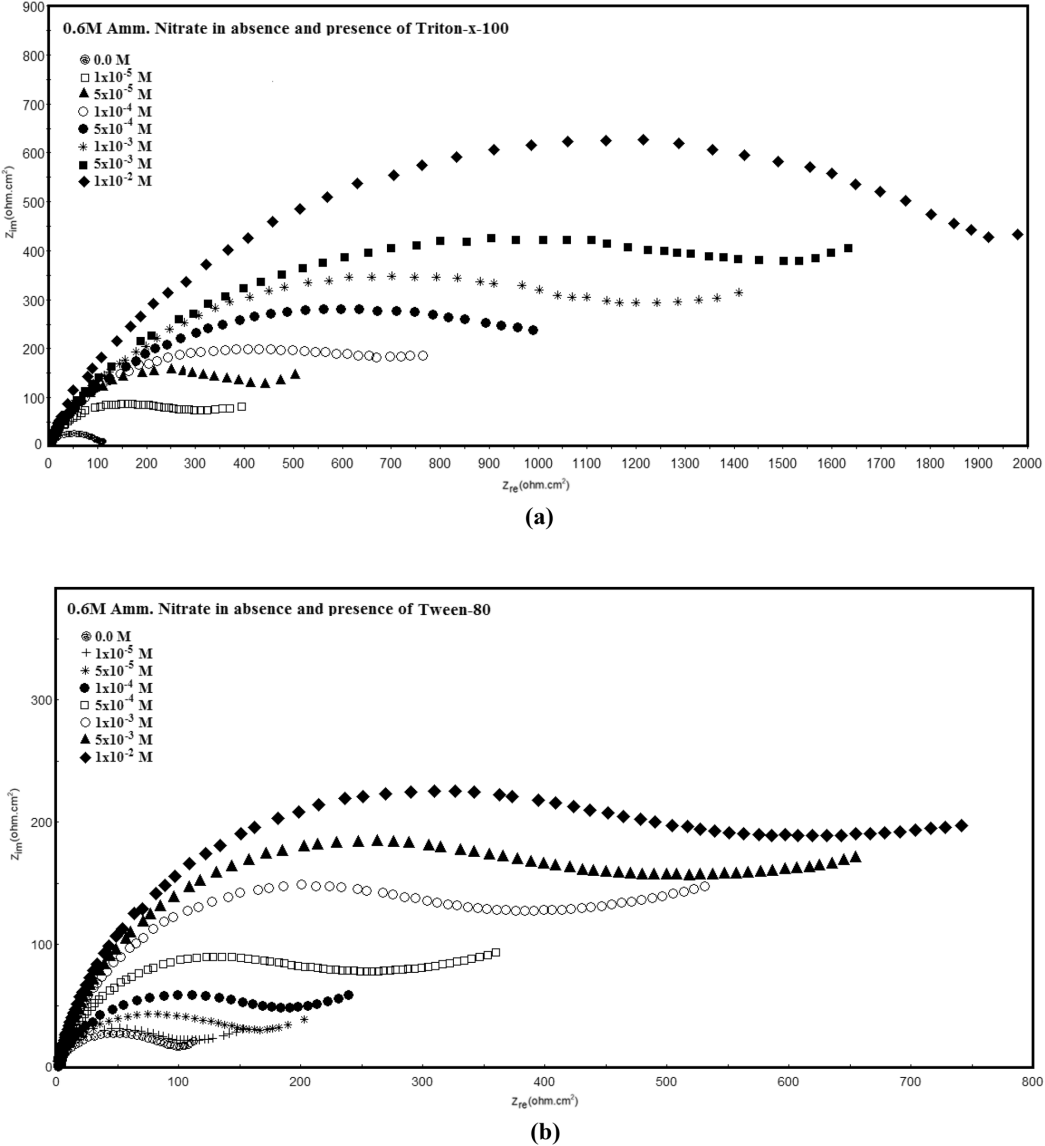
Nyquist plots of permanganate—phosphate coated steel in 0.6 M NH_4_NO_3_ solution (the coats contain different concentrations of each of Triton –x-100 (**a**) and Tween -80 (**b**) surfactants).

**Figure 7:**
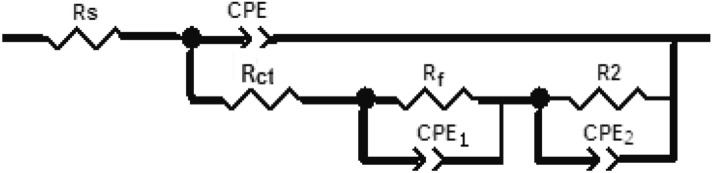
Schematic for the equivalent circuit of coated steel.

Nyquist impedance plots of permanganate—phosphate coated steel in 0.6 M NH_4_NO_3_, (the coats contain different concentrations of Triton-X-100 or Tween-80) are depicted in Fig. 6. These plots show three capacitive semicircles; the low-frequency semicircle is associated with charge transfer resistance and double layer capacity, while the high-frequency semicircle is associated with the behaviour of the conversion coat during the corrosion process. Fitting the experimental data to the equivalent circuit model is depicted in Fig. 7. The circuit includes R_s_ which represents the solution resistance; R_ct_ is the charge transfer resistance and CPE is the constant phase element related to the double-layer capacitance. The parallel combination of CPE2 and R2 are related to the capacitance and resistance of the conversion coat. The analysis of impedance spectra was carried out three times to avoid the percentage error of analysis.

The overlaid impedance spectra given in Fig. 6 and Table 2 show that the size of the semicircle and *R*_*ct*_ values increase with increasing the concentration of both of the two surfactants in the coating solution which suggests that both surfactants improve the resistances of the permanganate—phosphate coat against corrosion in 0.6 M NH_4_NO_3_ solution.

**Table 2:** Electrochemical impedance parameters of Permanganate -phosphate coated steel in 0.6 M NH_4_NO_3_ solution in the absence and presence of different concentrations of surfactants in the coating solution at 30 °C.

Type	Conc., mol/L	*R*_*s*_ (Ohm cm^2^)	*Q*_*f*_ (µF/cm)	*n*_1_	*R*_*p*_ (Ohm cm^2^)	*R*_*ct*_ (Ohm cm^2^)	Q_dl_ (µF/cm)	*n*_2_	*R*_2_ (Ohm cm^2^)	*Q*_3_ (µF/cm)	*n*_3_	% *P*
Triton-X-100	0	0.7	51	0.94	97	113	1326	0.93	216	4271	0.94	0
	1 × 10–5	0.9	50	0.93	146	294	1266	0.92	311	4199	0.91	61.6
	5 × 10–5	1.1	48	0.95	158	489	1101	0.84	400	4000	0.9	76.9
	1 × 10–4	1.0	47	0.93	184	699	910	0.91	523	3910	0.91	83.8
	5 × 10–4	1.0	45	0.84	206	987	824	0.93	671	3843	0.8	88.6
	1 × 10–3	0.9	43	0.93	232	1179	713	0.94	810	3705	0.9	90.4
	5 × 10–3	1.1	41	0.95	259	1438	622	0.84	988	3626	0.93	92.1
	1 × 10–2	1.0	38	0.94	278	1820	509	0.94	1011	3522	0.82	93.7
Tween 80	0	0.7	51	0.94	106	113	1326	0.93	216	4271	0.94	0
	1 × 10–5	1.1	49	0.83	133	129	1210	0.91	240	4112	0.82	12.4
	5 × 10–5	1.0	48	0.94	139	167	1011	0.94	294	3940	0.94	32.3
	1 × 10–4	1.0	46	0.92	146	226	914	0.8	352	3910	0.91	50
	5 × 10–4	0.9	45	0.83	176	299	821	0.91	465	3845	0.9	62.2
	1 × 10–3	1.2	44	0.94	205	411	702	0.9	521	3789	0.84	72.5
	5 × 10–3	1.1	42	0.93	243	592	644	0.82	641	3690	0.92	80.9
	1 × 10–2	1.0	39	0.82	262	712	543	0.93	866	3547	0.84	84.1

The information in Table 2 demonstrates that when the concentration of the two surfactants increases, the double-layer capacitance decreases (*Q*_*dl*_) which is attributed to the adsorption of the surfactant molecules on the steel surface. The protection efficiency of the coat (%* P*) was measured using the equation^42^:

$$\% P = [({\text{R}}_{{{\text{ct}}}} {-}{\text{R}}_{{{\text{ct}}}}^{{\text{o}}} )/{\text{R}}_{{{\text{ct}}}} ] \times {1}00$$

where *R*_*ct*_ and *R*_*ct*_*o* are the charge transfer resistances, in the presence and absence of surfactants respectively. The data in the table show that the protection efficiency of the coats increases with raising the concentration of the surfactants and reaches to 93.7% in presence of Triton- X-100 and 84.1% in presence of Tween 80 for 1 × 10^–2^ M of each surfactant.

**Figure 8:**
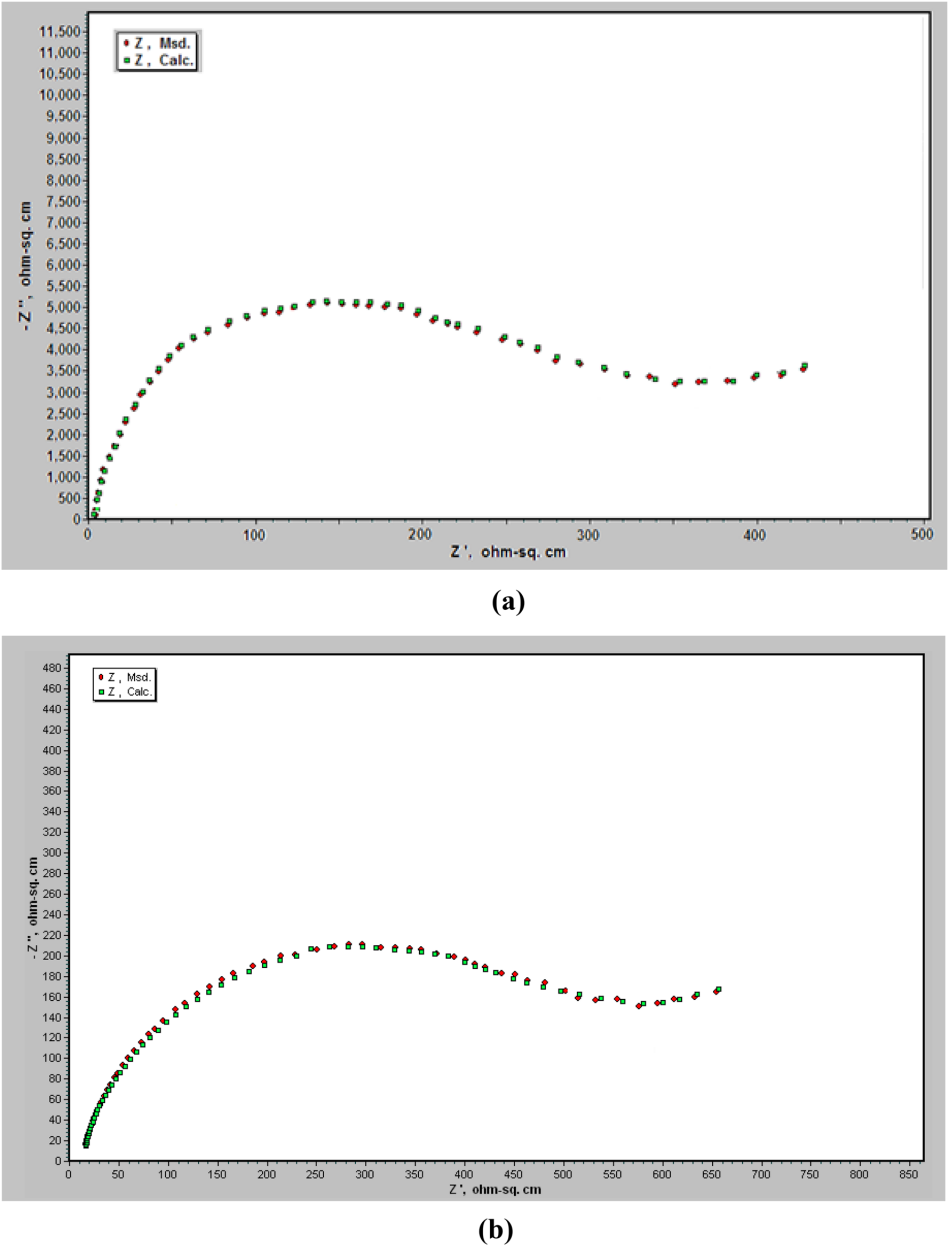
The computer fitting of the measured data for Nyquist plots in 0.6 M NH_4_NO_3_ solution for permanganate—phosphate coated steel (the coats contain (**a**): 1 × 10^−5^ M Triton –x-100 and (**b**): 5 × 10^−3^ M Tween -80 surfactants).

Figure 8 shows the computer fitting of the measured data for Nyquist plots in 0.6 M NH_4_NO_3_ solution for permanganate—phosphate coated steel (the coats contain (**a**): 1 × 10^−5^ M Triton –x-100 (**b**) and 5 × 10^−3^ M Tween -80 surfactants). Fitting the EIS data to the equivalent circuit model revealed a good agreement between fitted and measured spectra. The repeatability of all parameters data of measurements are about ± **0.001 to ± 0.18** and the error less than 3%.

**Figure 9:**
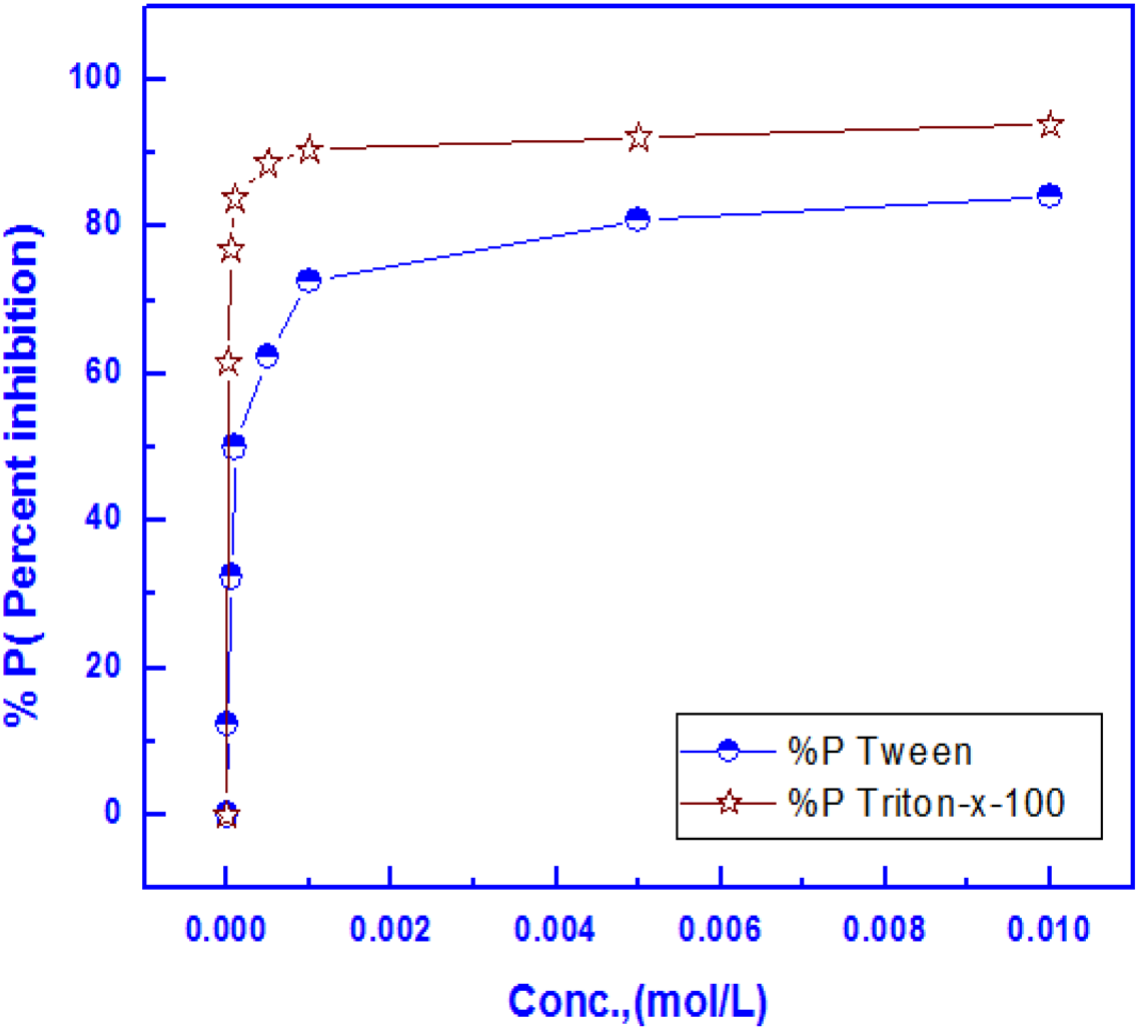
Effect of the concentration of Triton-X-100 and Tween-80 surfactants on the protection efficiency of the permanganate_phosphate coats in 0.6 M NH_4_NO_3_ solution.

Figure 9 shows the variation of the protection efficiency of the coats with concentration of each of surfactants in the coating bath. It is clear that Triton-X-100 has higher protection efficiency than Tween-80 which confirms the EDX results.

### Potentiodynamic polarization results

**Figure 10:**
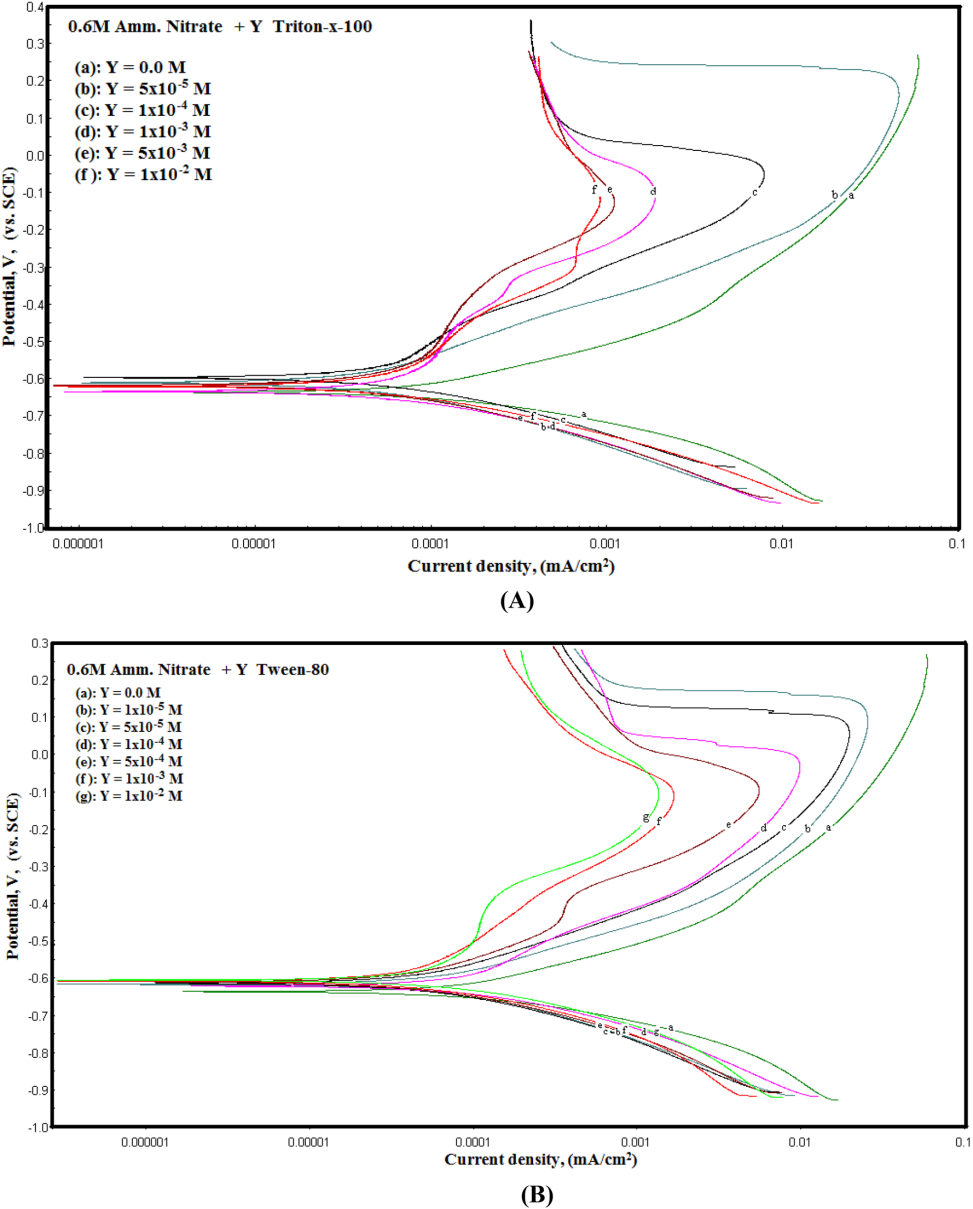
Polarization curves of permanganate—phosphate coated steel (the coats contain different concentration of surfactants) in 0.6 M NH_4_NO_3_ solution.

Figure 10a, b shows the potentiodynamic polarization curves of the permanganate—phosphate coated steel (the coats contain different concentrations of surfactants) in 0.6 M NH_4_NO_3_ solution. As the figure shows, the anodic portion of the polarization curves is affected rather than the cathodic one when Triton-X-100 or Tween-80 are added to the coating solution indicating that the two surfactants retard the oxidation reaction of the steel at the anodic sites of the surface. Also, it is obvious from the anodic part of the Fig. 10 that the polarization curves show activation behaviour followed by a decrease in the current density obviously at high potentials which means a formation of passive protective film on the steel surface. This passivation is mainly attributed to the formation of the conversion coat.

The electrochemical corrosion parameters including corrosion potential (E_corr_), corrosion current density (i_corr_), anodic (β_a_) and cathodic (β_c_) Tafel slopes were determined by extrapolation of the linear Tafel segments of the potentiodynamic polarization curves are presented in Table 3. The protection efficiency (%*P*) was computed as follows^43^:

$$\% P = \left[ {\left( {{\text{i}}_{0} {-}{\text{i}}} \right)/{\text{i}}_{0} } \right] \times {1}00$$

**Table 3:** The electrochemical parameters for the coated steel that was performed using a bath containing different concentrations of surfactants in the coating solution at 30 °C.

Type	Conc., mol/L	*−* *E*_*corr.*_ (mV vs. SCE)	*β*_*a*_	*−* *β*_*c*_	*i*_*corr*_*.* (μA cm^−2^)	*%P*
			(mV decade^−1^)			
Triton-X-100	0.0	641	176	141	15.05	0.0
	1 × 10^–5^	646	146	149	06.09	59.5
	5 × 10–^5^	654	134	154	03.85	74.4
	1 × 10^–4^	662	126	161	02.92	80.6
	5 × 10^–4^	670	117	169	02.12	85.9
	1 × 10^–3^	675	109	175	02.01	86.6
	5 × 10^–3^	680	101	179	01.55	89.7
	1 × 10^–2^	686	89	184	01.27	91.7
Tween-80	0.0	652	182	141	15.05	0.0
	1 × 10^–5^	643	152	145	13.34	11.4
	5 × 10–^5^	649	141	147	10.44	30.6
	1 × 10^–4^	656	133	149	07.87	47.7
	5 × 10^–4^	662	126	156	06.11	59.4
	1 × 10^–3^	669	117	161	04.72	68.6
	5 × 10^–3^	675	107	168	03.33	77.9
	1 × 10^–2^	679	94	172	02.72	81.9

The tabulated data shows that the i_corr_ values decrease and %*P* values increase with the increase of the concentrations of both surfactants. Tables 2 and 3 show that there is a good agreement between the values of %*P* obtained from the impedance and potentiodynamic polarization for the two surfactants.

### Porosity of coat

Permanganate-phosphate conversion coating is mainly composed of insulating hopeites; the pores in the coat are generally regarded as the exposed area of the substrate, which can be measured from the results-obtained from the potentiodynamic polarization and electrochemical impedance spectrophotometry techniques^44,45^. Using the following equation^46^:

$$\% {\text{ Porosity}} = \left( {{\text{R}}_{{{\text{ps}}}} /{\text{ R}}_{{\text{p}}} } \right){1}0^{{ - (}{{\text{DEcorr}}/\upbeta {\text{a}})}} \times {1}00$$

where R_ps_ and R_p_ are the polarization resistances of the bare and coated steel samples, respectively. ΔE_corr_ is the difference between the corrosion potentials of the bare and coated steel samples. β_a_ is referred to slope of the anodic Tafel line derived from the polarization curves. The porosity values of the permanganate-phosphate coat on the steel surface in absence and presence of different concentrations of Triton-X-100 or Tween-80 surfactants were calculated using the electrochemical data presented in Tables 2 and 3 and given in Table 4.

**Table 4:** The electrochemical parameters used in the determination of the porosity of permanganate-phosphate coat on steel in the absence and presence of different concentrations of each of Triton-X-100 or Tween-80 in the coating bath.

Type	Conc., mol/L	− *E*_*corr*._ ( mV vs. SCE)	*β*_*a*_ (mV decade^−1^)	*R*_*p*_	% Porosity
Triton-X-100	0 (Bare metal)	698	182	21	–
	0 (coated metal)	641	176	97	39.6
	1 × 10^–5^	646	146	146	25.9
	5 × 10^–5^	654	134	158	24.5
	1 × 10^–4^	662	126	184	19.4
	5 × 10^–4^	670	117	206	15.3
	1 × 10^–3^	675	109	232	13.2
	5 × 10^–3^	680	101	259	10.8
	1 × 10^–2^	686	89	278	8.9
Tween 80	0 (coated metal)	652	182	106	35.5
	1 × 10^–5^	643	152	133	36.3
	5 × 10^–5^	649	141	139	33.7
	1 × 10^–4^	656	133	146	29.6
	5 × 10^–4^	662	126	176	23
	1 × 10^–3^	669	117	205	18.1
	5 × 10^–3^	675	107	243	14.2
	1 × 10^–2^	679	94	262	12.7

**Figure 11:**
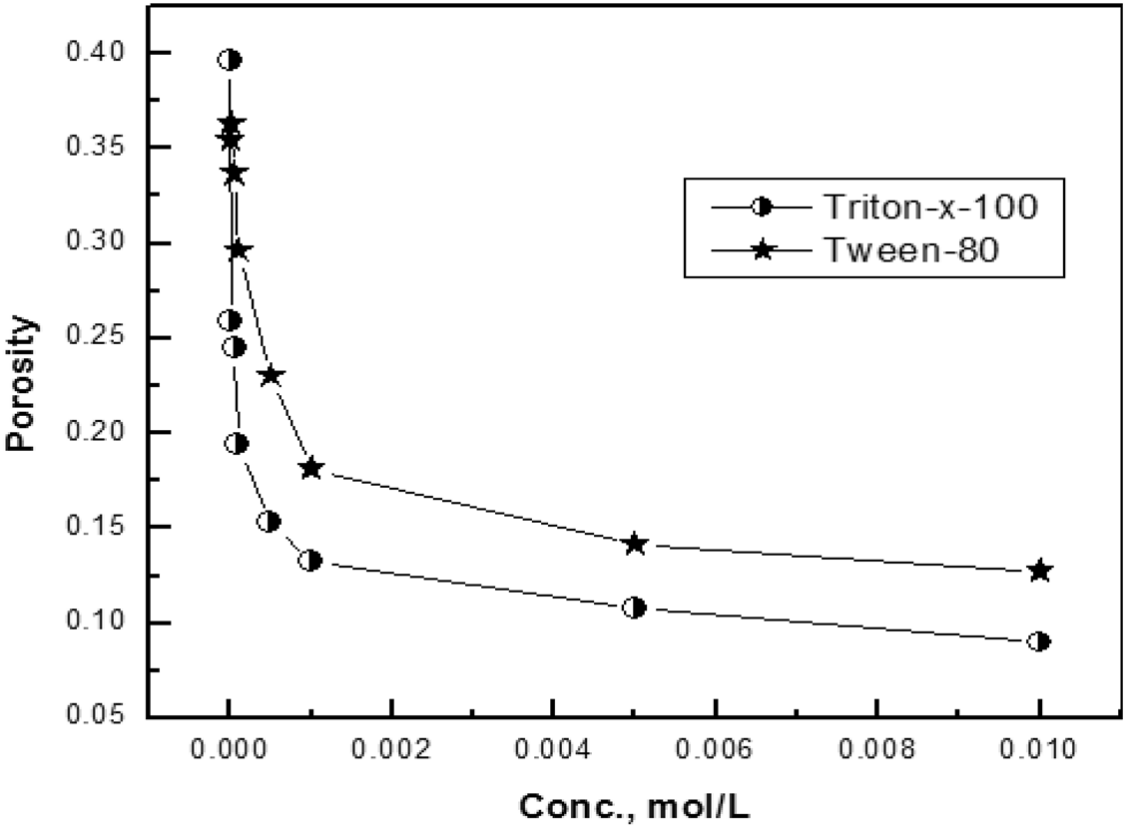
Relation between the porosity of the coat and concentration of each of Triton-X-100 and Tween-80 present in the coating bath.

Variation of the porosity of the coat with the concentration of each of Triton-X-100 and Tween-80 are presented in Fig. 11. It is clear that, in presence of low concentrations (< 0.001 M) of the surfactants, in the coating bath porosity of the coat sharply decrease. However, in presence of higher concentrations of the surfactant in the solution, it has a slight effect on the porosity of the coat. this behaviour confirms the EDX results.

### Thickness of the coat

Steel coupons with area 2 cm^2^ rectangular mild steel and with the same chemical composition of steel samples used in the electrochemical measurements were used in the experiment. The thickness of the permanganate-phosphate-coats for varied concentration of the two surfactants are shown in Table 5. It is clear that the thickness of the coat drops as the concentration of surfactant raised, which indicates that the presence of the surfactant molecules in the coating bath enhancing the coating process making the coat less porous. The results show also that in presence of Triton-X-100 the thicknesses of the coats are less than those in presence of Tween-80 which confirm the results of SEM, EDX and porosity.

**Table 5:** The thickness values of the conversion-coated steel samples in presence of different concentration of each of Triton-X-100 and Tween-80 in their coating bath.

Type	Conc., mol/L	Thickness of the coat (µm)
Triton-X-100	0	0.429
	5 × 10–5	0.426
	5 × 10–4	0.423
	5 × 10–3	0.42
	1 × 10–2	0.416
Tween 80	5 × 10–5	0.428
	5 × 10–4	0.425
	5 × 10–3	0.423
	1 × 10–2	0.42

### Coat stability

**Figure 12:**
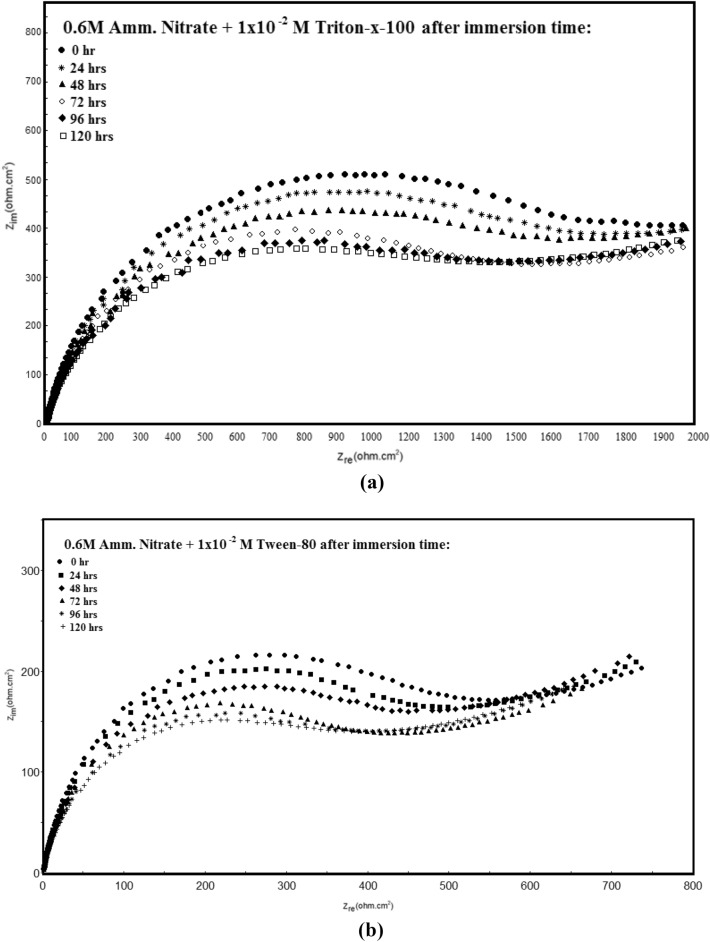
Nyquist plots of Permanganate -phosphate coated steel (the coats contain 1 × 10^–2^ M Triton –x-100 (**a**), and 1 × 10^–2^ M Tween -80 (**b**)) in 0.6 M NH_4_NO_3_ solution at different immersion times at 30 °C.

Figure 12 shows the Nyquist diagrams of the permanganate- phosphate coated steel (the coats contain 1 × 10^–2^ M Triton –x-100 or 1 × 10^–2^ M Tween -80) in 0.6 M NH_4_NO_3_ at different immersion times. As can be observed, the capacitive semicircle's size gradually shrinks as immersion duration increases. The previously utilized equivalent circuit (Fig. 7) was used to evaluate these graphs. The electrochemical parameters values obtained through EIS for Permanganate -phosphate coated steel at different immersion times are given in Table 6.

**Table 6:** Electrochemical impedance parameters of Permanganate -phosphate coated steel (the coats contain 1 × 10^–2^ M Triton –x-100 or 1 × 10^–2^ M Tween -80) in 0.6 M NH_4_NO_3_ solution at different immersion times at 30 °C.

Type	Immersion time (hrs)	*R*_*s*_ (Ohm cm^2^)	*Q*_*f*_ (µF/cm)	*n*_1_	R_p_ (Ohm cm^2^)	*R*_*ct*_ (Ohm cm^2^)	Q_dl_ (µF/cm)	*n*_2_	R_2_ (Ohm cm^2^)	Q_3_ (µF/cm)	*n*_3_	*% P*
Triton-X-100	0	1	38	0.94	278	1820	509	0.93	1011	3522	0.94	93.79
	24	0.9	40	0.93	273	1818	512	0.93	1009	3533	0.91	93.78
	48	0.8	43	0.91	271	1815	516	0.9	1001	3537	0.9	93.77
	72	0.9	44	0.89	269	1811	519	0.91	994	3543	0.92	93.76
	96	0.9	44	0.87	267	1807	523	0.93	989	3549	0.91	93.74
	120	0.8	46	0.86	265	1803	527	0.91	976	3554	0.91	93.73
Tween 80	0	1	39	0.94	262	712	543	0.93	866	3547	0.94	84.12
	24	0.8	41	0.92	259	708	546	0.9	858	3557	0.9	84.03
	48	0.8	42	0.9	257	704	549	0.91	851	3562	0.9	83.94
	72	0.9	44	0.89	253	701	554	0.93	844	3571	0.91	83.88
	96	0.8	45	0.88	251	696	559	0.92	841	3577	0.93	83.76
	120	1	47	0.86	247	691	564	0.92	836	3581	0.9	83.64

**Figure 13:**
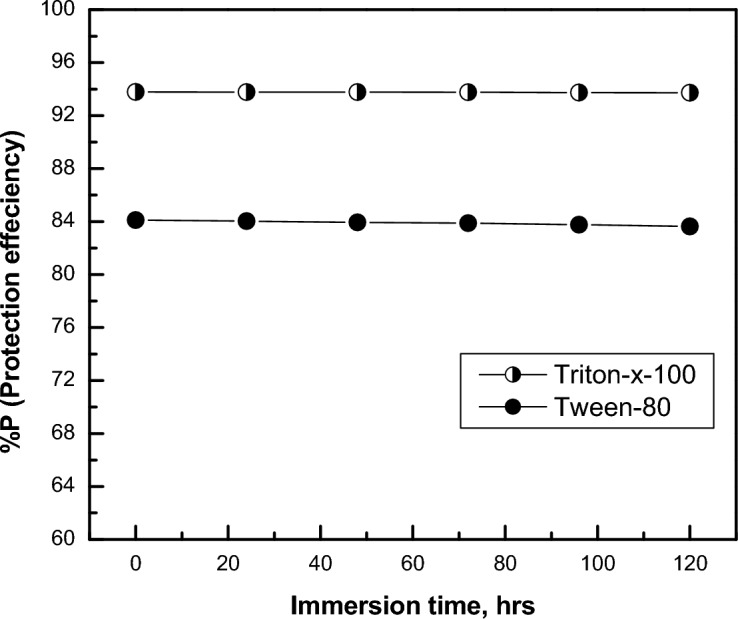
Effect of immersion time (in NH_4_NO_3_ solution) of the permanganate- phosphate coats contains 1 × 10^–2^ M Triton–x-100 or 1 × 10^–2^ M Tween-80 on their protection efficiency at 30 °C.

Figure 13 shows the dependence of the protection efficiency of the coat (% *P*) on the immersion time of permanganate_phosphate coated steel contains 1 × 10^–2^ M of surfactants in 0.6 M NH_4_NO_3_ solution. It is clear that increasing immersion time up to 120 h, leads to a minor decrease in the protection efficiency, demonstrating the remarkable stability of the Permanganate_phosphate coated steel.

### XPS results

**Figure 14:**
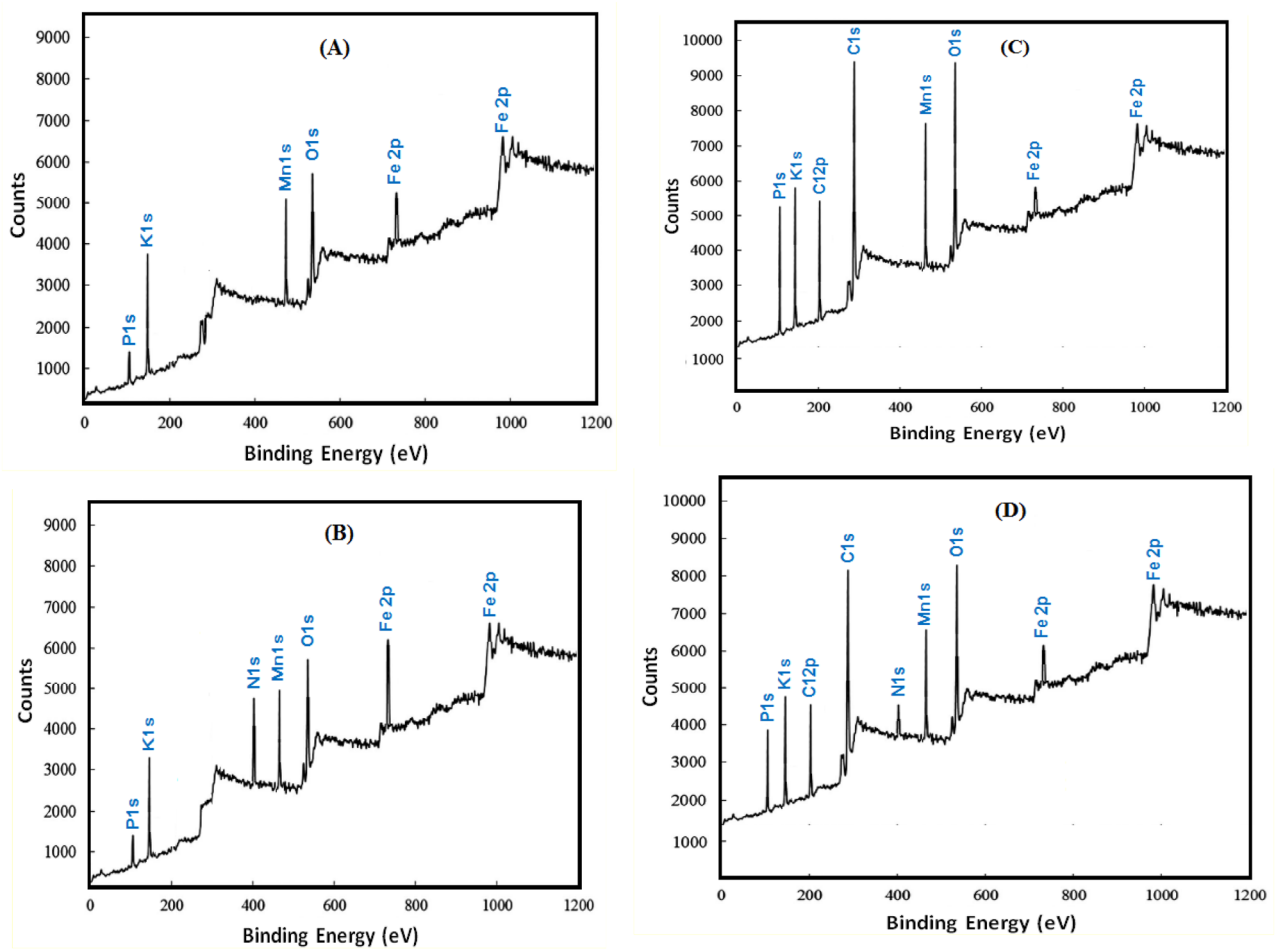
Full XPS spectra for: (**A**): coated steel, (**B**): coated steel after 12 h immersion in 0.6 M NH_4_NO_3_ (**C**): coated steel its coating bath contains 1 × 10^–2^ M Triton-X-100 after immersion in 0.6 M NH_4_NO_3_ for 12 h and (**D**) Coated steel its coating bath contains 1 × 10^–2^ M Tween-80 after immersion in 0.6 M NH_4_NO_3_ for 12 h.

Figure 14 shows the full XPS spectra of (A) coated steel free from surfactants, (B) coated steel free from surfactants after immersion in 0.6 M NH_4_NO_3_ for 12 h, (C) coated steel its coating bath contains 1 × 10^–2^ M Triton-X-100 after immersion in 0.6 M NH_4_NO_3_ for 12 h, (D) coated steel its coating bath contains 1 × 10^–2^ M Tween-80 after immersion in 0.6 M NH_4_NO_3_ for 12 h.

Table 7 summarizes the results in this Fig. 14 and represents the intensities of the peak of the different elements in the four samples.

**Table 7:** Intensity of the peaks of the elements in the coats and coat after immersion in NH_4_NO_3_ solution for 12 h.

Element binding energy eV	Intensity of the peak			
	Coat (A)	Coat after immersion in NH_4_NO_3_ (B)	Coat contains 10^−2^ M Triton-X-100 after immersion (C)	Coat contains 10^−2^ M Tween-80 after immersion (D)
**P1s** 156.5	1300	700	3300	2100
**K1s** 179.6	3800	2100	3800	2700
**C12p** 206.7	–	–	3100	2500
**C1s** 300	–	–	7000	5500
**N1s** 408.7	–	1900	–	800
**Mn1s** 488.7	3400	1800	3500	2500
**O1s** 561.3	2600	2000	5500	4000
**Fe2p** 766.8	1700	1100	500	1000
**Fe2p** 998.6	1900	1500	1100	2000

The XPS results of the coated steel free from surfactants (Fig. A) show six peaks at 156.2, 179.6, 488.7, 561.3, 766.8 and 998.6 eV. These peaks correspond to the elements and their respective chemical states present on the surface of the coated steel.

After immersion in NH_4_NO_3_ solution for 12 h (Fig. B) a new peak appeared at 408.7 eV which corresponds to N1s state of nitrate. This indicates that the corrosive anions adsorbed and reacted with the coating forming a layer of the corrosion products on the surface. When Triton-X-100 is added to the coating bath (Fig. C) two new peaks appeared at 206.7 and 300 eV correspond to C1s and C12p of the surfactant. This indicates that the surfactant is incorporated into the coating layer. Additionally, the peak intensities of the P, K and Mn increased indicating that the presence of Triton-X-100 in the coat increased its protection efficiency, The peak of N is disappeared which indicates that the presence of the surfactant in the coating bath prevent the adsorption of the (NO_3_^−^) ion at the steel surface due to its high absorbability. However, when Tween-80 is added (Fig. D) to the coating bath, the two carbon peaks appeared with low intensities indicating that this surfactant is less efficient than Triton-X-100. And the nitrogen peak appeared which indicates that (NO_3_^−^) anions are adsorbed and reacted with the coat due to the weak absorbability of the Tween-80 molecules.

### Mechanism of action of the surfactants in the conversion coating process

**Figure 15:**
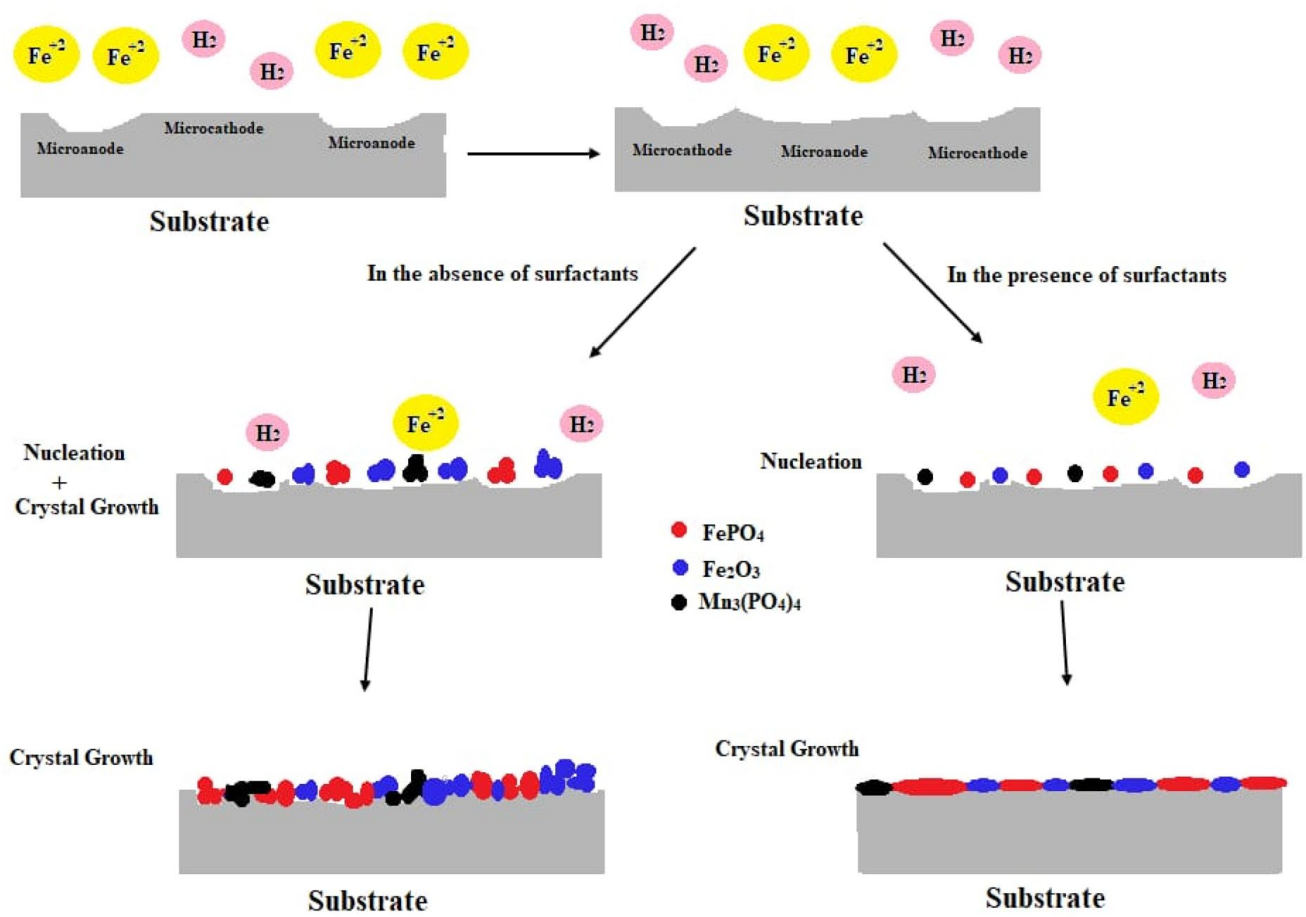
Schematic representation of the mechanism of conversion coating.

A complex series of chemical and electrochemical processes occur at the steel/solution interface in the coating bath. The formation of the coat mainly takes place through the following steps which represented in Fig. 15^47–49^.

### Electrochemical dissolution of steel

Steel instantly forms a microgalvanic couple when submerged in the acidic conversion coating bath. The dissolution of iron is done through the following reaction at the microanodes.

$${\text{Fe}} = {\text{ Fe}}^{{{2} + }} 2{\text{e}}^{ - }$$

The dissolved Fe^2+^ is speedily oxidized to Fe^3+^ by the potassium permanganate in the conversion coating bath

$${\text{Fe}}^{{{2} + }} = {\text{Fe}}^{{{3}+}} + {\text{e}}^{ - }$$

The hydrogen evolution occurs simultaneously at the microcathodes was done via the following reaction:

$$2{\text{H}}^{ + } + 2{\text{e}}^{ - } = {\text{H}}_{{{2}({\text{g}})}}$$

## Deposition of insoluble salts at steel surface

An amorphous film of both oxides and phosphates of iron is deposited

$${\text{Fe}}^{{{3} + }} + {\text{PO}}4^{{{3} - }} = {\text{ FePO}}_{{4}}$$

$$2{\text{Fe}}^{{{3} + }} + 3{\text{H}}_{{2}} {\text{O }} = {\text{ Fe}}_{{2}} {\text{O}}_{{3}} + 6{\text{H}}^{ + }$$

Also, it is reported that The Mn species were mostly existing as Mn^4+^ in the coating^28^.

$${\text{Mn}}^{{{4} + }} + {\text{ PO4}}^{{{3} - }} = {\text{ Mn}}_{{3}} \left( {{\text{PO}}_{{4}} } \right)_{{4}}$$

## Growth of the deposited coat

The deposition of both FePO_4_ and Fe_2_O_3_ on the steel surface is done via two steps: nucleation and crystal growth. The deposited coat behaviour is varied according to whether the coat is formed in the presence or the absence of surfactants. The surfactants play a weighty influence in the microstructure of the deposited coat.

- In the absence of the surfactants

The rate at which iron dissolves is high, the high concentration of Fe^2+^ in the conversion coating bath allows the high rate of the crystal growth of the deposited salts over the nucleation process. The insoluble salts will be deposited fast and random which leads to the formation of loosely high porous coat with lower steel substrate protection effectiveness.

b. In the presence of the surfactants

Adsorption of surfactants on the steel surface retarding the dissolution of metal, the low concentration of Fe^2+^ in the conversion coating bath allows higher rate of nucleation process over the crystal growth and so a uniform coat is formed. This mechanism is confirmed by measuring the coat thickness and porosity of the coats in the absence and presence of the surfactants and by the XPS results.

## Conclusion

1. Electrochemical, SEM, EDX and XPS results indicated that the presence of each of Triton-X-100 or Tween-80 surfactants in the coating bath of the permanganate-phosphate coat effectively improved its corrosion resistance.
2. EIS results demonstrated that the presence of 0.01 M of each of Triton-X-100 or Tween-80 in the coating solution increased the protection efficiency of the coat up to 93.0% and up to 84.1%, respectively. The greater of that in presence of Triton-X-100 is believed to the presence of a benzene ring in its molecular structure.
3. A mechanism for the action of the surfactants in the coating process is proposed using the findings of the different techniques.

## Data Availability

All the data presented in the current study are available from the corresponding author on reasonable request.
